# High‐dose post‐transplant cyclophosphamide impairs γδ T‐cell reconstitution after haploidentical haematopoietic stem cell transplantation using low‐dose antithymocyte globulin and peripheral blood stem cell graft

**DOI:** 10.1002/cti2.1171

**Published:** 2020-09-23

**Authors:** Nicolas Stocker, Béatrice Gaugler, Myriam Labopin, Agathe Farge, Yishan Ye, Laure Ricard, Eolia Brissot, Remy Duléry, Simona Sestili, Giorgia Battipaglia, Clémence Médiavilla, Annalisa Paviglianiti, Anne Banet, Zoe Van De Wyngaert, Tounes Ledraa, Mohamad Mohty, Florent Malard

**Affiliations:** ^1^ INSERM, Centre de Recherche Saint‐Antoine (CRSA) Sorbonne Université Paris France; ^2^ Service d’Hématologie Clinique et Thérapie Cellulaire Hôpital Saint‐Antoine, AP‐HP Paris France; ^3^ Acute Leukemia Working Party Paris Study Office European Society for Blood and Marrow Transplantation Paris France; ^4^ Bone Marrow Transplantation Center The First Affiliated Hospital School of Medicine Zhejiang University Hangzhou China

**Keywords:** allogeneic haematopoietic cell transplantation, Epstein–Barr virus, haploidentical haematopoietic cell transplantation, immune reconstitution, post‐transplantation cyclophosphamide, γδ T cell

## Abstract

**Objectives:**

Haploidentical haematopoietic cell transplantation (Haplo‐HCT) using peripheral blood stem cell (PBSC) grafts and post‐transplant cyclophosphamide (PTCy) is being increasingly used; however, data on immunological reconstitution (IR) are still scarce.

**Methods:**

This retrospective study evaluated T‐cell immunological reconstitution in 106 adult patients who underwent allogeneic haematopoietic cell transplantation for haematologic malignancies between 2013 and 2016.

**Results:**

At D30, while conventional T cells reached similar median counts in Haplo‐HCT recipients (*n* = 19) and controls (*n* = 87), γδ and Vδ2^+^ T‐cell median counts were significantly lower in Haplo‐HCT recipients and it persists at least until D360 for Vδ2^+^ T cells. PTCy induces a significant reduction in early γδ and Vδ2^+^ T‐cell proliferation at D  7. At one year, the rate of increase in Epstein–Barr virus (EBV) viral load was significantly higher in Haplo‐HCT recipients as compared to controls (61% versus 34%, *P* = 0.02). In multivariate analysis, a higher γδ T‐cell count (> 4.63 μL^−1^) at D30 was the only independent parameter significantly associated with a reduced risk of increase in EBV viral load (RR 0.34; 95% CI, 0.15–0.76, *P* = 0.009).

**Conclusion:**

Immunological reconstitution of γδ T cells is significantly delayed after Haplo‐HCT using PTCy and low‐dose ATG and is associated with an increased risk of increase in EBV viral load.

## Graphical abstract

In this study, immune reconstitution analyses with flow cytometry observed an impaired γ/δ and Vδ2^+^ T‐cell reconstitution following haploidentical haematopoietic cell transplantation using peripheral blood stem cell grafts and post‐transplant cyclophosphamide (PTCy). PTCy induces a significant reduction in early proliferation of γ/δ and Vδ2^+^ T‐cell subsets while impaired γ/δ T‐cell recovery was associated with an increased risk of increase in Epstein–Barr virus load.

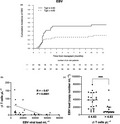

## Introduction

Allogeneic haematopoietic stem cell transplantation (Allo‐HCT) is the only curative treatment for many patients with malignant and non‐malignant diseases. Nevertheless, a fully matched related donor is available for only 30% of patients. Therefore, the use of alternative donors, matched or mismatched unrelated donors, umbilical cord blood or haploidentical donors, was developed. Haploidentical haematopoietic cell transplantation (Haplo‐HCT) is associated with an important bidirectional alloreactivity, and first results were disappointing with a very high toxicity and mortality of the procedure.^1^ Fortunately, development of Haplo‐HCT using post‐transplant cyclophosphamide (PTCy) significantly improved the rates of graft‐versus‐host disease (GvHD), non‐relapse mortality and engraftment.^2^, ^3^, ^4^, ^5^, ^6^, ^7^ Compared with Allo‐HCT with matched related or unrelated donor, retrospectives studies reported similar overall survival (OS) and disease‐free survival (DFS) with Haplo‐HCT and even sometimes a decreased incidence of acute and/or chronic GvHD.^2^, ^3^, ^4^, ^5^, ^6^, ^7^ PTCy acts through induction of functional impairment of alloreactive T cells without toxic effects on haematopoietic stem cells.^8^ Furthermore, after allogeneic stimulation, donor regulatory T cells express a higher level of aldehyde dehydrogenase, the enzyme responsible for *in vivo* detoxification of cyclophosphamide. Therefore, donor regulatory T cells are resistant to PTCy‐induced cytotoxicity and contribute to the prevention of GvHD after PTCy.^1^

Nevertheless, we and others recently reported high incidences of viral reactivation following Haplo‐HCT with PTCy.^9^, ^10^, ^11^ In particular, an increased EBV viral load was reported in 19–53% of patients^9^, ^10^, ^12^ and up to 72% when a longer follow‐up of 2 years was considered.^9^

Several studies have shown the role of gamma delta (γδ) T cells in EBV surveillance. De Paoli *et al*.^13^ have described an expansion of γδ T cells during the acute phase of EBV‐induced infectious mononucleosis, suggesting their possible role in controlling EBV infection. Furthermore, pamidronate‐expanded Vγ9^+^Vδ2^+^ T cells can directly kill EBV‐lymphoblastoid cell lines *in vitro* and *in vivo*.^14^ γδ T cells exhibit both innate and adaptive features^15^ and differ from conventional α/β T cells not only in TCR composition, but also in their development, tissue distribution and physiological functions.^16^, ^17^, ^18^ The Vδ2^+^ T‐cell subset is the predominant peripheral blood γδ T‐cell population in most adults.^19^

In the setting of Allo‐HCT, expansion of γδ Vδ1^+^ T cells has been reported in one patient with an increase in EBV viral load after an umbilical cord blood transplantation.^20^ Furthermore, inverse correlations between CD4/CD8 double negative or γδ Vδ2^+^ T‐cell recovery and increase in EBV viral load were reported after Haplo‐HCT with high‐dose antithymocyte globulin (ATG).^21^, ^22^

With this background, we evaluated the impact of PTCy administration on γδ T‐cell immune reconstitution after Allo‐HCT with peripheral blood stem cell (PBSC) grafts, and the correlations with the increase in EBV viral load.

## Results

### Characteristics of patients, donors and transplantations

For cohort 1, patients, donors and transplant characteristics are summarised in Table 1. Twenty‐nine patients had a matched related and 58 a matched unrelated donor (control group, *n* = 87), while 19 patients received Haplo‐HCT with PTCy (PTCy group). The median age was 56 years (range, 18–70) in the control and 57 (range, 27–71) in the PTCy group (*P* = 0.83). All donor/recipient pairs were EBV positive (respectively, positive/negative 2%, negative/positive 10% and positive/positive 87% in the control group versus 16%, 0% and 84% in the PTCy group).

**Table 1:** Cohort 1 population and transplant characteristics

Characteristics	Control group (*n* = 87)	PTCy group (*n* = 19)	*P*‐value
Patient age in years, median (range)	56 (18–70)	57 (27–71)	0.83
Patient gender			
Male	53 (61)	15 (79)	0.14
Female	34 (39)	4 (21)	
Donor gender			
Female to male	22 (25)	8 (42)	0.14
CMV‐seronegative donor–recipient pair	25 (29)	4 (21)	0.50
EBV serostatus donor–recipient pair			
Positive/Negative	2 (2)	3 (16)	**0.03**
Negative/Positive	9 (10)	0	
Positive/Positive	76 (87)	16 (84)	
Negative/Negative	0	0	
Diagnosis			
Myeloid malignancies	58 (67)	12 (63)	0.77
Acute myeloid leukaemia	28 (33)	10 (53)	
Myeloproliferative neoplasms	22 (25)	1 (5)	
Myelodysplastic syndrome	8 (9)	1 (5)	
Lymphoid malignancies	29 (33)	7 (37)	
Acute lymphoblastic leukaemia	17 (19)	4 (21)	
Non‐Hodgkin lymphoma	10 (12)	3 (16)	
Hodgkin lymphoma	2 (2)	0	
Disease Risk Index			
Low	2 (2)	0	0.78
Intermediate	44 (51)	8 (42)	
High	34 (39)	10 (53)	
Very High	7 (8)	1 (5)	
Conditioning regimen			
RIC	14 (16)	0	0.08
RTC	57 (66)	13 (68)	
Sequential	16 (18)	6 (32)	
Antithymocyte globulin			
Median (range)	5 (5–6)	5 (2.5–5)	0.07
2.5 mg kg^‐1^	0	3	
5 mg kg^‐1^	86	16	
6 mg kg^‐1^	1	0	
Post‐transplant immunosuppression			
CsA alone	15 (17)	0	**< 0.0001**
CsA and MMF	64 (74)	0	
CsA and MTX	8 (9)	0	
CsA and MMF and PTCy	0	19^a^ (100)	
Median follow‐up, months (range)	35 (22–42)	30 (19–41)	0.62

CMV, cytomegalovirus; CsA, cyclosporine A; EBV, Epstein–Barr virus; MMF, mycophenolate mofetil; MTX, methotrexate; PTCy, post‐transplant cyclophosphamide; RIC, reduced intensity conditioning; RTC, reduced toxicity conditioning; yrs, years.

Bold denotes statistically significant.

^a^ PTCy 50 mg kg^−1^ day^−1^ at D3 and D5 (2 patients received PTCy at D3 only).

According to the Disease Risk Index,^23^ patients were considered to be low, intermediate, high or very high risk (respectively, 2%, 51%, 39% and 8% in the control group and 0%, 42%, 53% and 5% in the PTCy group, *P* = 0.78). Twenty‐two patients who were not in complete remission at the time of Allo‐HCT, 16 being in the control (18%) and 6 being in the PTCy group (32%), received a sequential conditioning regimen, while the remaining patients received a RIC (respectively, 14 and 0) or a RTC regimen (respectively, 57 and 13; *P* = 0.08).

### Immune reconstitution

**Figure 1:**
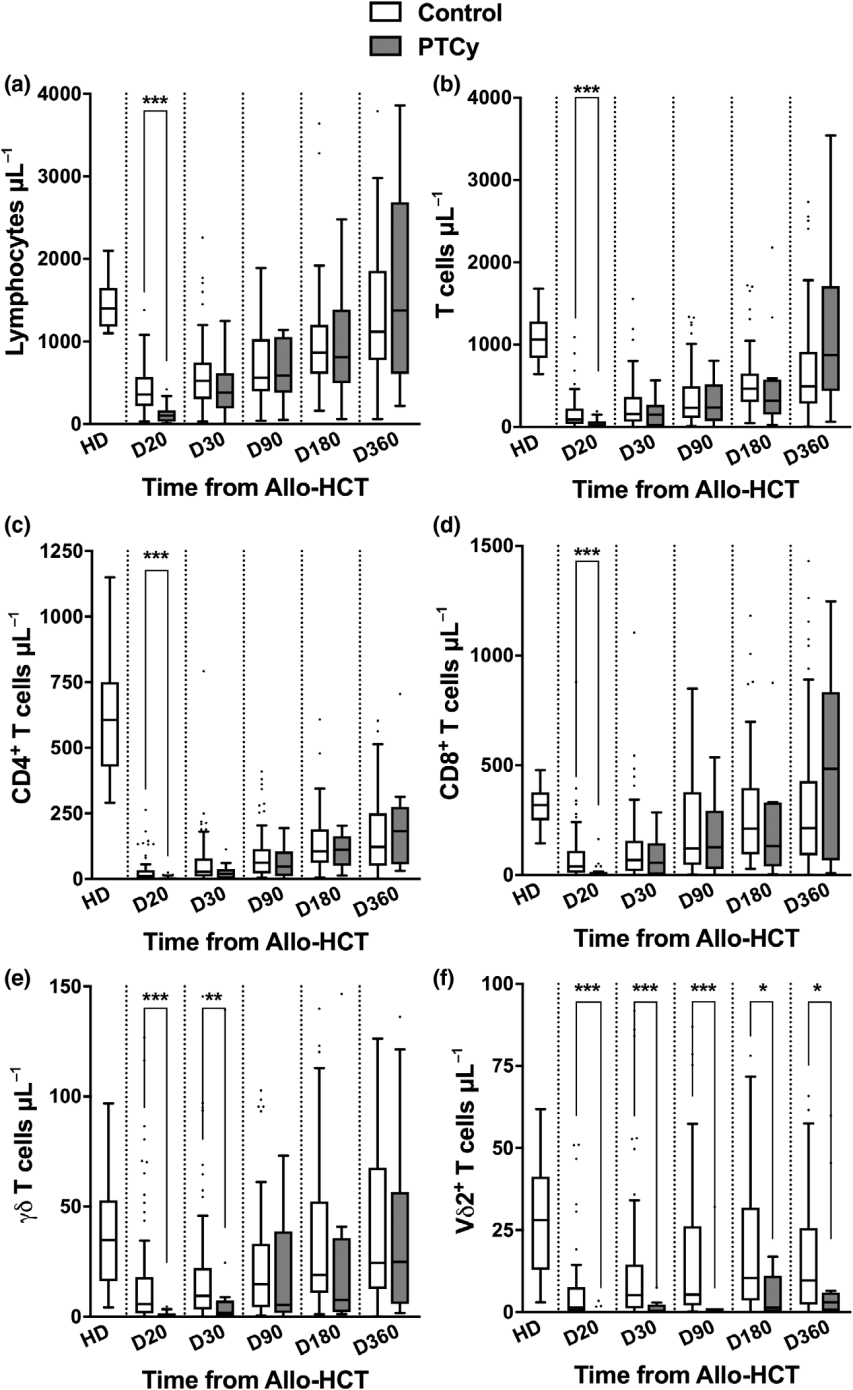
Immune recovery of T cells after Allo‐HCT. Kinetics of absolute lymphocyte **(a)**, T‐cell **(b)**, CD4^+^ T‐cell **(c)**, CD8^+^ T‐cell **(d)**, γδ T‐cell **(e)** and Vδ2^+^ T‐cell **(f)** counts in blood from control (*n* = 87) and Haplo‐HCT with PTCy (*n* = 19) recipients following Allo‐HCT. Samples from healthy donors serve as baseline reference. Box and whisker plots displaying the median, the 25th percentile, the 75th percentile of the distribution (box), and the most extreme data point (whiskers), which is no more than 1.5 times the interquartile range from the box. Allo‐HCT, allogeneic haematopoietic stem cell transplantation; HD, healthy donors; PTCy, post‐transplant cyclophosphamide.

The IR of T‐cell and γδ T‐cell subsets is illustrated in Figure 1 and summarised in Supplementary table 1. At D20 after Allo‐HCT, the median counts of all immune cell subsets were significantly lower in PTCy groups as compared to the control group (Figure 1). At D30, total lymphocytes, CD3^+^ T cells, CD4^+^ T cells and CD8^+^ T cells reached similar median counts in the two groups (Figure 1a–d). In contrast, γδ T‐cell and Vδ2^+^ T‐cell median counts remained significantly low at D30 (Figure 1e, f). Furthermore, while γδ T‐cell median count in the PTCy group reached values similar to those in the control at D90 (Figure 1e), Vδ2^+^ T‐cell median counts remained significantly lower in the PTCy than the control group up to D360 after transplantation (Figure 1f). Finally, compared to healthy donors, Vδ2^+^ T‐cell counts remained significantly lower in both PTCy and control groups at least until D360 (Figure 1f). Of note, IR of T‐cell and γδ T‐cell subsets was similar in a subgroup analysis including only patients receiving CsA and MMF (Supplementary figure 2). In contrast, acute GvHD and subsequent use of corticosteroids had no impact on γδ T‐cell and Vδ2^+^ T‐cell immune recovery (Supplementary figure 3) but were associated with an impaired long‐term immune reconstitution of lymphocytes, CD3^+^ T cells, CD4^+^ T cells and CD8^+^ T cells (Supplementary figure 3).

### Early proliferation of T cells

**Figure 2:**
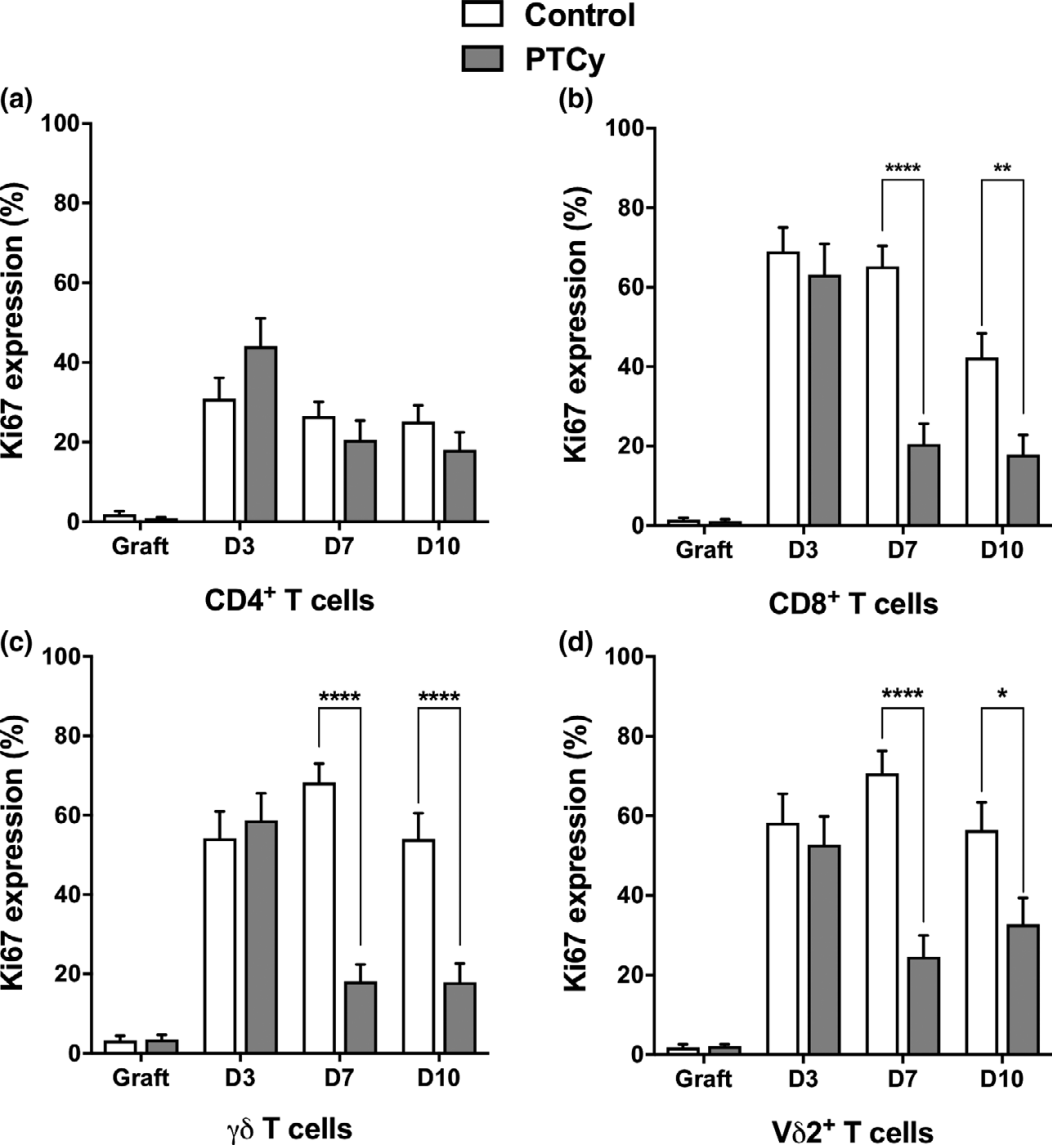
Kinetics of Ki67‐expressing T cells after Allo‐HCT. Kinetics of Ki67‐expressing CD4^+^ T‐cell **(a)**, CD8^+^ T‐cell **(b)**, γδ T‐cell **(c)** and Vδ2^+^ T‐cell **(d)** proportions in the graft and at D3, D7 and D10 in control (not receiving PTCy, *n* = 16) and Haplo‐HCT with PTCy (*n* = 12) recipients after Allo‐HCT. Bars displaying the mean of Ki67 MFI and 95% confidence intervals (error bars) are shown. Allo‐HCT, allogeneic haematopoietic stem cell transplantation; PTCy, post‐transplant cyclophosphamide.

To decipher the mechanism of impaired γδ and Vδ2^+^ T‐cell recovery after Haplo‐HCT with PTCy, we analysed T‐cell proliferation (Ki67^+^), on fresh samples of the PBMC graft, and at early time points after Allo‐HCT (D3, D7 and D10) in a second cohort of 16 controls and 12 PTCy recipients. Patient, donor and transplant characteristics are summarised in Supplementary table 2 for cohort 2. In the graft, the proportions of Ki67‐expressing cells were low for all T‐cell subsets (Figure 2). Graft infusion was associated with proliferation of all T‐cell subsets, as demonstrated by the high proportion of Ki67‐expressing cells at D3 in CD8^+^ T cells, γδ T cells, Vδ2^+^ T cells and to a lesser extent CD4^+^ T cells. Interestingly, there were no differences at D3 (before administration of PTCy) in the proportions of Ki67‐expressing T cells between control and PTCy groups (Figure 2).

After PTCy treatment, at D7 and D10 after Allo‐HCT, we observed a significant decrease in Ki67‐expressing CD8^+^, γδ and Vδ2^+^ T cells (Figure 2b–d) in the PTCy group, as compared with the control group. In contrast, there were no differences in the proportions of Ki67‐expressing CD4^+^ T cells between Haplo‐HCT and control recipients at D7 and at D10 (Figure 2a).

### Clinical outcomes

Clinical outcomes were only evaluated in cohort 1. The median follow‐up among surviving patients was 33 months (range, 22–42). At 2 years, the rates of NRM, PFS, GPFS and OS were 9%, 72%, 64% and 78%, respectively (Table 2). At D180, the cumulative incidences (CIs) of grade II‐IV and grade III‐IV aGvHD were 20% and 2%, respectively. The 2‐year CIs of cGvHD, extensive cGvHD and relapse were 36%, 14% and 19%, respectively, while CIs of CMV reactivation and increase in EBV viral load at one year were 27% and 39%, respectively. There were no differences between the control group and the PTCy group with respect to all outcomes considered, except for increase in EBV viral loads at 12 months (Table 2): this was significantly higher in the PTCy group (respectively, 61% versus 34%, *P* = 0.02).

**Table 2:** Clinical outcomes after allogeneic haematopoietic stem cell transplantation

Outcomes	All patients (*n* = 106)	Control group (*n* = 87)	PTCy group (*n* = 19)	*P*‐value
aGvHD incidence at D180, % (95% CI)				
Grade II–IV	20 (12–29)	19 (11–29)	24 (7–46)	0.59
Grade III–IV	2 (0–6)	2 (1–8)	0	0.52
cGvHD incidence at month 24, % (95% CI)	36 (26–45)	36 (26–46)	33 (13–55)	0.70
Extensive	14 (7–19)	12 (6–19)	17 (4–37)	0.67
Non‐relapse mortality at month 24, % (95% CI)	9 (4–15)	8 (4–15)	11 (2–30)	0.70
CMV incidence at month 12, % (95% CI)	27 (18–37)	24 (14–35)	46 (15–72)	0.16
EBV incidence at month 12, % (95% CI)	39 (30–49)	34 (24–45)	61 (34–80)	**0.02**
Relapse incidence at month 24, % (95% CI)	19 (12–27)	19 (12–28)	19 (4–42)	0.61
Progression‐free survival at month 24, % (95% CI)	72 (64–81)	73 (63–82)	70 (47–93)	0.83
GPFS at month 24, % (95% CI)	64 (54–74)	64 (53–75)	62 (37–87)	0.72
Overall survival at month 24, % (95% CI)	78 (70–86)	78 (69–87)	79 (61–97)	0.85
Median follow‐up, months (range)	33 (22–42)	35 (22–42)	30 (19–41)	0.62

aGvHD, acute graft‐versus‐host disease; cGvHD, chronic graft‐versus‐host disease; CI, confidence interval; CMV, cytomegalovirus; EBV, Epstein–Barr virus; GPFS, graft‐versus‐host disease and progression‐free survival; PTCy indicate post‐transplantation cyclophosphamide.

Bold denotes statistical significance.

**Figure 3:**
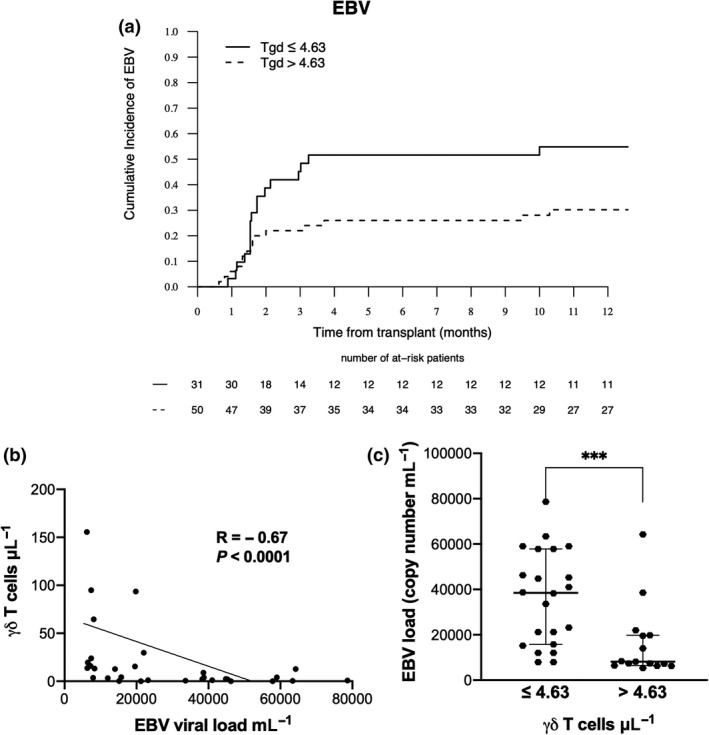
Increase in EBV viral load and γδ T‐cell counts at D30 after Allo‐HCT. Probability of increase in EBV viral load according to γδ T‐cell count (below or over the best value of 4.63 μL^−1^) at D30 after Allo‐HCT **(a)**. Correlation between increase in EBV viral load and γδ T‐cell count at D30 after Allo‐HCT **(b)**. Increase in EBV viral load according to γδ T‐cell count (below or over the best value of 4.63 μL^−1^) at D30 after Allo‐HCT **(c)**. The median of EBV viral load and 95% confidence intervals (error bars) are shown. Allo‐HCT, allogeneic haematopoietic stem cell transplantation; EBV, Epstein–Barr virus.

To further investigate a potential role of T cells in the increased risk of increase in EBV viral load after Haplo‐HCT with PTCy, we then performed ROC curves analysis for each T‐cell subset. It revealed that only γδ T‐cell and Vδ2^+^ T‐cell counts at D30 following Allo‐HCT were accurate discriminators of the risk of increase in EBV viral load. Patients having the lowest γδ and Vδ2^+^ T‐cell counts (respectively, ≤ 4.63 μL^−1^ and ≤ 0.49 μL^−1^; the cut‐off values used to segregate the patients between low and high γδ and Vδ2^+^ T‐cell counts) at D30 had a significantly higher risk of increase in EBV viral load (respectively, *P* = 0.006 and *P* = 0.003) (Supplementary table 3 and Figure 3a). In contrast, γδ and Vδ2^+^ T‐cell counts at D30 had no impact on the main outcomes after Allo‐HCT, including relapse, NRM and OS (data not shown). In multivariate logistic regression analysis, a higher γδ T‐cell count (> 4.63 μL^−1^) at D30 following Allo‐HCT was the only independent parameter significantly associated with a reduced risk of increase in EBV viral load (RR 0.34; 95% CI, 0.15–0.76, *P* = 0.009) (Table 3). Finally, in the subgroup of patients with an increase in EBV viral load (*n* = 45), median EBV viral copy number was 15748 (range, 5192–580 000) copies mL^−1^. Interestingly, we find a correlation between EBV viral copy number and γδ T cell (*R* = −0.67, *P* < 0.0001; Figure 3b), median EBV viral copy number being significantly lower in patients with higher γδ T‐cell count (> 4.63 μL^−1^) at D30 (median 8116 copies mL^−1^ versus 38 502 copies mL^−1^, *P* = 0.0008; Figure 3c).

**Table 3:** Multivariate analysis of EBV risk factors

Covariates	Hazard ratio (95% CI)	*P*‐value
RIC vs RTC/sequential	0.39 (0.11–1.3)	0.13
Haplo‐HCT vs Control	1.01 (0.39–2.6)	0.99
High–very‐high DRI vs others	1.09 (0.52–2.3)	0.82
γδ T cells > 4.63 μL^−1^ vs ≤ 4.63 μL^−1^ at D30	0.34 (0.15–0.76)	**0.009**

CI, confidence interval; DRI, disease risk index; Haplo‐HCT, haploidentical haematopoietic stem cell transplantation; RIC, reduced intensity conditioning; RTC, reduced toxicity conditioning; vs, versus.

Bold denotes statistical significance.

## Discussion

A delayed IR after Allo‐HCT has been associated with higher risk of relapse,^24^ NRM,^24^, ^25^ GvHD^25^ and bacterial, fungal and viral infections.^26^ Therefore, IR is essential to control transplant complications and improve survival after Allo‐HCT.^17^

In the setting of Allo‐HCT with PTCy, previous analyses of IR focused on α/β T cells.^27^, ^28^, ^29^ It was shown in animal models that T memory stem cells derive from naïve T cells after Allo‐HCT.^28^ In humans, Kanakry *et al*.^27^ reported a favorable CD4^+^, CD8^+^ and CD4^+^CD25^+^Foxp3^+^ T‐cell reconstitution after Allo‐HCT with bone marrow grafts and PTCy as a single agent for GvHD prophylaxis. Furthermore, it was shown that CMV seropositivity can drive T‐cell reconstitution and α/β T‐cell receptor diversity.^29^ Data on γδ T cells are scarcer. Retiere *et al*.^30^ recently reported that the use of PTCy was associated with fewer γδ T cells early after Allo‐HCT compared to ATG, while it was the opposite for α/β T cells.

The current study shed a new light on γδ T‐cell reconstitution and its impact on the increased EBV viral load after Haplo‐HCT with PTCy and PBSC grafts. Administration of PTCy and low‐dose ATG was associated with an early profound lymphopenia: at D20, all lymphocyte subsets assessed were significantly decreased compared to patients receiving ATG alone. Thereafter, α/β CD4^+^ and CD8^+^ T cells progressively increased, and at D30, levels in the PTCy group were similar to those of patients receiving ATG alone, as previously described.^30^ In contrast, γδ and Vδ2^+^ T‐cell IR was very slow and significantly delayed in patients receiving PTCy, with a dramatically lower median counts of Vδ2^+^ T cells up to one year after Haplo‐HCT. Dose of ATG was consistent across patients, with the majority of patients (96%) receiving 5 mg kg^−1^ ATG; therefore, we were not able to evaluate the impact of ATG dose on IR and patients’ outcomes.

Since PTCy induces alloreactive proliferative T‐cell dysfunction and suppression,^8^ we assessed the effect of PTCy on the proliferation capacity of the different T‐cell subsets to understand the mechanism of the profound γδ T‐cell deficiency observed after PTCy. Interestingly, the proportions of proliferating CD8^+^, γδ and Vδ2^+^ T cells at D3 after Allo‐HCT and before PTCy administration were high and comparable to those of patients receiving ATG alone. After PTCy administration, we observed a deleterious impact of PTCy on the CD8^+^, γδ and Vδ2^+^ T‐cell proliferation, while proliferation was not affected in the control group.

Nevertheless, while CD8^+^ T cells rapidly reached similar median counts, γδ T‐cell counts remained significantly lower, up to one year for Vδ2^+^ T cells, after Haplo‐HCT and PTCy. Aldehyde dehydrogenase (ALDH), the major mechanism of resistance to cyclophosphamide, has been reported to drive regulatory T‐cell resistance to PTCy.^27^ Therefore, we assessed if the deleterious impact of PTCy on CD8^+^, γδ and Vδ2^+^ T cells was related to a lower expression of ALDH in those cells, but we could not find any difference between cell subsets within the PBSC graft (data not shown). Interestingly, we previously reported that PBSC grafts contain more than 10 times fewer γδ T cells than CD8^+^ T cells,^31^ and we hypothesised that the different pool size of CD8^+^ and γδ T cells in the PBSC graft may explain the differences in IR.

A meta‐analysis recently reported that high γδ T‐cell values after Allo‐HCT were associated with less disease relapse, fewer infections and higher OS and disease‐free survival.^32^ Nevertheless, despite a significantly delayed γδ T‐cell IR in patients receiving an Haplo‐HCT with PTCy and ATG, there was no difference in relapse incidence, OS and PFS between the two groups, suggesting that γδ T cells are not critical for disease control in this setting of Haplo‐HCT. In contrast, as previously reported,^9^ the incidence of increased EBV viral load was significantly higher in patients receiving Haplo‐HCT with low‐dose ATG and PTCy compared to patients with matched donor and receiving low‐dose ATG alone, being 61% versus 34%, respectively (*P* = 0.02). Furthermore, in multivariate analysis, the impact of PTCy was overcome by a low number of γδ T cells at D30, which was the only parameter associated with a higher incidence of increased EBV viral load [HR, 0.34 (0.15–0.76), *P* = 0.009]. This finding is in accordance with previous studies which reported a correlation between the Vδ2^+^ T cell or CD4/CD8 double‐negative T‐cell recovery and the occurrence of increase in EBV viral load in the setting of Haplo‐HCT with high‐dose ATG.^21^, ^22^

Our study has several limitations, in particular regarding the absence of external validation cohort and the heterogeneity of the population in terms of conditioning regimen, GvHD prophylaxis or haematological diseases. In an attempt to have a more homogeneous population, we included only patients receiving a fludarabine and busulfan‐based conditioning regimen; nevertheless, we cannot exclude that various doses of busulfan and additional drugs administered in some patients, in particular thiotepa, may have influence IR. Regarding GvHD prophylaxis, our findings were similar in a subgroup analysis of patients receiving CsA and MMF. Finally, we must also highlight that while γδ T cells represent only a minor part of the circulating T cells (1–5%), they are present in much higher number within epithelial tissues (10–100%).^33^ It would be therefore very important to evaluate whether γδ T‐cell infiltration correlates with our finding in the peripheral blood and the role of γδ T‐cell homoeostasis in antiviral immunity.

## Conclusion

Overall, the current study shows that administration of PTCy after Haplo‐HCT can induce a profound and prolonged γδ T‐cell depletion. Furthermore, we report an inverse correlation of the recovery of Vδ2^+^ T cells with increase in EBV viral load following Haplo‐HCT using PTCy and low‐dose ATG. This result is in line with the previous observations reported after Haplo‐HCT using the GIAC protocol.^22^ Manipulation of PBSC grafts, based on Vδ2^+^ T‐cell sorting and *ex vivo* culture with bisphosphonates,^21^ followed by a delayed administration of autologous Vδ2^+^ T cells could be a new cellular therapy to improve the prophylaxis of increase in EBV viral load in the Haplo‐HCT with PTCy setting.

## Methods

### Patients

This retrospective single‐centre study included a cohort of 106 patients who underwent Allo‐HCT for haematological malignancies between April 2013 and December 2016 at the Saint‐Antoine University Hospital (AP‐HP, Paris, France). All patients who received Allo‐HCT with low dose of ATG and a granulocyte colony‐stimulating factor mobilised PBSC graft from a matched related or unrelated donor (control group) or an haploidentical donor with PTCy (PTCy group) and with at least one frozen peripheral blood sample available at day + 30 for IR analysis were included. A group of 10 healthy donors [HD group, Etablissement Français du Sang (EFS, Paris Saint‐Antoine‐Crozatier, Paris, France)] was established to serve as baseline reference [median age 45 (range, 32–61) years, 70% were male]. In addition, a second independent cohort of 28 Allo‐HCT patients was used to prospectively analyse early T‐cell proliferation in fresh samples. Written informed consent was obtained from each patient. This study was approved by the hospital’s institutional review board and the local ethics committee (Comité de Protection des Personnes Ile de France X, reference 29‐2016).

### Transplantation procedure

All patients received the preparative regimen as in‐patients in private rooms, and remained hospitalised until haematopoietic and clinical recovery. They all received granulocyte colony‐stimulating factor mobilised PBSC as grafts and *in vivo* T‐cell depletion with low‐dose ATG [thymoglobulin (Sanofi, France) 5 mg kg^−1^ median dose (range, 2.5–6)]. In addition, patients with an haploidentical donors received standard PTCy (50 mg kg^−1^ day^−1^) at D3 and D5. Patients with refractory disease received a sequential conditioning regimen based on thiotepa 5–10 mg kg^−1^, etoposide 400  mg m^−2^ and cyclophosphamide 1600 mg m^−2^ between days (D) ‐15 and ‐9, followed by, after a 3‐day rest, reduced intensity conditioning (RIC) with fludarabine 150 mg m^−2^ and intravenous busulfan 6.4 mg kg^−1^ on D‐6 to D‐1,^34^ while other patients received a RIC or a reduced toxicity conditioning (RTC) regimen based on 150 mg m^−2^ fludarabine, 6.4–9.6 mg kg^−1^ day^−1^ busulfan with or without 5 mg kg^−1^ thiotepa. Post‐transplantation immunosuppression consisted of cyclosporine A (CsA) alone for matched sibling donors, CsA and mycophenolate mofetil (MMF) for matched unrelated donor and haploidentical donors, or CsA and a short course of methotrexate (MTX) for patients with a major ABO mismatch incompatibility. CsA was administered intravenously at a 3 mg kg^−1^ day^−1^ dosage starting on D‐2 or D‐3, and changed to twice daily oral dosing as soon as tolerated.^35^, ^36^ MMF was administered at a fixed oral dose of 2 g day^−1^ starting from D5 without adjustment. MTX was administered at 15 mg m^−2^ on D1 and 10 mg m^−2^ on D3 and D6. In the absence of GvHD, MMF and CsA were tapered over 4 weeks starting from D60 and D90, respectively. Supportive care was the same for all patients during the whole study period. Empiric broad‐spectrum antibiotics were administered if a patient developed a temperature ≥ 38.3°C, or ≥ 38°C for 1 h, or showed any sign of infection. Anti‐infectious prophylaxis consisted of acyclovir while trimethoprim–sulfamethoxazole or atovaquone was started after neutrophil recovery for one year and pursued beyond if CD4^+^ T cells remained below 0.2 × 10^9^ cells L^−1^. Cytomegalovirus (CMV), Epstein–Barr virus (EBV), human herpesvirus 6 (HHV6) and *Toxoplasma gondii* were routinely screened by quantitative polymerase chain reaction (PCR) until at least D100. For CMV reactivation, preemptive ganciclovir or foscavir was initiated when CMV was above 1000 international units mL^−1^. For the increase in EBV viral load in blood, preemptive rituximab was given when EBV was above 5000 international units mL^−1^. Acute GvHD (aGvHD) was diagnosed and graded according to the revised Glucksberg criteria,^37^ and chronic GvHD (cGvHD) was diagnosed and graded according to the Seattle standard criteria.^38^

### Flow cytometry

Peripheral blood samples were collected at D20, D30, D90, D180 and D360 following Allo‐HCT. Peripheral blood mononuclear cells (PBMCs) were isolated using a Lymphocyte Separation Medium (MP Biomedicals, Illkirch‐Graffenstaden, France) by density gradient centrifugation, and an aliquot was cryopreserved for storage. After thawing, PBMCs were stained with the following antibody panel: anti‐TCR Vβ11 (FITC), anti‐CD4 (PE), anti‐TCR γδ (PECy5.5), anti‐CD56 (AA700), anti‐CD3 (AA750), anti‐TCR Vδ2 (PB) and anti‐CD8 (KRO) (Beckman Coulter, Villepinte, France); anti‐CD161 (PE/Dazzle 594), anti‐TCR Vα24 (PC7) and anti‐TCR Vα7.2 (APC) (BioLegend, Ozyme, Saint‐Quentin en Yvelines, France); and anti‐CD45 (BV605) and anti‐CD16 (BV786) (BD Biosciences, Le Pont‐de‐Claix, France). For the analysis of proliferation, fresh PBMCs from the PBSC graft and from Allo‐HCT recipients (at D3, D7 and D10 after Allo‐HCT) were isolated as above and stained with the following antibody panel: anti‐CD4 (PE), anti‐TCR γδ (PECy5.5), anti‐CD56 (AA700), anti‐CD3 (AA750), anti‐TCR Vδ2 (PB) and anti‐CD8 (KRO) (Beckman Coulter); anti‐CD161 (PE Vio770) (Miltenyi, Bergisch‐Gladbach, Germany); anti‐TCR Vα7.2 (APC) (BioLegend); anti‐CD45 (BV605) (BD Biosciences) followed by Ki67 analysis, after permeabilisation and incubation with anti‐Ki67 (FITC) (BD Biosciences). Data were acquired on a Cytoflex flow cytometer (Beckman Coulter) and analysed using Kaluza Analysis v2.1 software (Beckman Coulter). To calculate absolute values of cells per microlitre of blood, the number of lymphocytes on routine clinical blood counts from the same day was collected from the patient charts. Based on these parameters, we calculated the proportions of cells in the blood of the patients for each cell population analysed by flow cytometry. Gating strategies used to identify innate‐like lymphoid cells are described in Supplementary figure 1.

### Statistical analysis

Differences between categorical and continuous variables were carried out using the Fisher and the Mann–Whitney tests, respectively. The cumulative incidence of aGvHD, cGvHD, viral reactivations, relapse and non‐relapse mortality (NRM) was estimated, and groups were compared using Gray’s test. Relapse was defined as the competitive risk for NRM, and NRM was defined as the competing risk for relapse. Progression‐free survival (PFS) was calculated from the date of Allo‐HCT until the time to relapse or progression. OS was calculated from the date of Haplo‐HCT until the time of death or the last observation if a patient remained alive. Refined GvHD‐free and progression‐free survival (GPFS) was defined as a combination of survival with no evidence of relapse/progression, grade III–IV aGvHD and extensive cGvHD.^39^ Probabilities of PFS, GPFS and OS were estimated using the Kaplan–Meier method, and groups were compared using the log‐rank test. The cut‐off values of cell concentrations at D30 after Allo‐HCT were assessed by constructing ROC curves and then calculating the area under the curve (AUC) for each one in order to identify the best cut‐off value on the whole cohort. Cox proportional hazard models were used to compare incidences of increase in EBV viral load and included relevant variables differing between the groups (*P* ≤ 0.10). For these analyses, only serial samples collected before aGvHD and relapse were considered. All tests were 2‐sided. The type I error rate was fixed at 0.05. Statistical analyses were performed with SPSS 24.0 (SPSS Inc, Chicago, IL, USA) and R 3.4.0 [R Core Team (2017). R: A language and environment for statistical computing. R Foundation for Statistical Computing, Vienna, Austria. URL https://www.R‐project.org/.].

## Conflict of interest

Mohamad Mohty reports grants and/or lecture honoraria from Janssen, Sanofi, MaaT Pharma, JAZZ pharmaceutical, Celgene, Amgen, BMS, Takeda, Pfizer and Roche, all outside the submitted work. Florent Malard reports lecture honoraria from Therakos/Mallinckrodt, Biocodex, Janssen, Keocyt, Sanofi, JAZZ pharmaceutical and Astellas, all outside the submitted work. Rémy DULERY reports lecture honoraria from Keocyt, Sanofi and Novartis, all outside the submitted work. The other authors declare no competing financial interests.

## Author contributions

**Nicolas Stocker:** Conceptualization; Formal analysis; Investigation; Methodology; Writing‐original draft. **Béatrice Gaugler:** Conceptualization; Formal analysis; Investigation; Methodology; Supervision; Validation; Writing‐review & editing. **Myriam Labopin:** Formal analysis; Writing‐review & editing. **Agathe Farge:** Investigation; Writing‐review & editing. **Yishan Ye:** Investigation; Writing‐review & editing. **Laure Ricard:** Investigation; Writing‐review & editing. **Eolia Brissot:** Investigation; Writing‐review & editing. **Remy Dulery:** Investigation; Writing‐review & editing. **Simona Sestili:** Investigation; Writing‐review & editing. **Giorgia Battipaglia:** Investigation; Writing‐review & editing. **Clémence Médiavilla:** Investigation; Writing‐review & editing. **Annalisa Paviglianiti:** Investigation; Writing‐review & editing. **Anne Banet:** Investigation; Writing‐review & editing. **Zoe Van De Wyngaert:** Investigation; Writing‐review & editing. **Tounes Ledraa:** Investigation; Writing‐review & editing. **Mohamad Mohty:** Conceptualization; Funding acquisition; Methodology; Project administration; Resources; Validation; Writing‐review & editing. **Florent Malard:** Conceptualization; Formal analysis; Investigation; Methodology; Supervision; Validation; Writing‐original draft; Writing‐review & editing.

## References

[1] Kanakry CG , Fuchs EJ , Luznik L . Modern approaches to HLA‐haploidentical blood or marrow transplantation. Nat Rev Clin Oncol 2016; 13: 10–24. 26305035 10.1038/nrclinonc.2015.128 PMC4695979

[2] Lorentino F , Labopin M , Fleischhauer K *et al* The impact of HLA matching on outcomes of unmanipulated haploidentical HSCT is modulated by GVHD prophylaxis. Blood Adv 2017; 1: 669–680. 29296709 10.1182/bloodadvances.2017006429 PMC5727822

[3] Ruggeri A , Sun Y , Labopin M *et al* Post‐transplant cyclophosphamide versus anti‐thymocyte globulin as graft‐ versus‐host disease prophylaxis in haploidentical transplant. Haematologica 2017; 102: 401–410. 32354866 10.3324/haematol.2020.247296 PMC8168508

[4] Law AD , Salas MQ , Lam W *et al* Reduced‐intensity conditioning and dual T lymphocyte suppression with antithymocyte globulin and post‐transplant cyclophosphamide as graft‐versus‐host disease prophylaxis in haploidentical hematopoietic stem cell transplants for hematological malignancies. Biol Blood Marrow Transplant 2018; 24: 2259–2264. 30009980 10.1016/j.bbmt.2018.07.008 PMC7110605

[5] Dreger P , Sureda A , Ahn KW *et al* PTCy‐based haploidentical vs matched related or unrelated donor reduced‐intensity conditioning transplant for DLBCL. Blood Adv 2019; 3: 360–369. 30723110 10.1182/bloodadvances.2018027748 PMC6373757

[6] Ahmed S , Kanakry JA , Ahn KW *et al* Lower graft‐versus‐host disease and relapse risk in post‐transplant cyclophosphamide‐based haploidentical versus matched sibling donor reduced‐intensity conditioning transplant for hodgkin lymphoma. Biol Blood Marrow Transplant 2019; 25: 1859–1868. 31132455 10.1016/j.bbmt.2019.05.025 PMC6755039

[7] Passweg JR , Baldomero H , Bader P *et al* Hematopoietic SCT in Europe 2013: recent trends in the use of alternative donors showing more haploidentical donors but fewer cord blood transplants. Bone Marrow Transplant 2015; 50: 476–482. 25642761 10.1038/bmt.2014.312 PMC4387247

[8] Wachsmuth LP , Patterson MT , Eckhaus MA , Venzon DJ , Gress RE , Kanakry CG . Post‐transplantation cyclophosphamide prevents graft‐versus‐host disease by inducing alloreactive T cell dysfunction and suppression. J Clin Investig 2019; 129: 2357–2373. 30913039 10.1172/JCI124218 PMC6546453

[9] Mohty R , Brissot E , Battipaglia G *et al* Infectious complications after post‐transplantation cyclophosphamide and anti‐thymocyte globulin‐based haploidentical stem cell transplantation. Br J Haematol 2019; 187: e64–e68. 31487392 10.1111/bjh.16189

[10] Crocchiolo R , Bramanti S , Vai A *et al* Infections after T‐replete haploidentical transplantation and high‐dose cyclophosphamide as graft‐versus‐host disease prophylaxis. Transpl Infect Dis 2015; 17: 242–249. 25648539 10.1111/tid.12365 PMC7169814

[11] Slade M , Goldsmith S , Romee R *et al* Epidemiology of infections following haploidentical peripheral blood hematopoietic cell transplantation. Transpl Infect Dis 2017; 19: e12629. 10.1111/tid.12629 PMC5459579 28030755

[12] Tischer J , Engel N , Fritsch S *et al* Virus infection in HLA‐haploidentical hematopoietic stem cell transplantation: incidence in the context of immune recovery in two different transplantation settings. Ann Hematol 2015; 94: 1677–1688. 26055139 10.1007/s00277-015-2423-y

[13] De Paoli P , Gennari D , Martelli P , Cavarzerani V , Comoretto R , Santini G . γδ T cell receptor‐bearing lymphocytes during Epstein‐Barr virus infection. J Infect Dis 1990; 161: 1013–1016. 2324529 10.1093/infdis/161.5.1013

[14] Xiang Z , Liu Y , Zheng J *et al* Targeted activation of human Vγ9Vδ2‐T cells controls Epstein‐Barr virus‐induced B cell lymphoproliferative disease. Cancer Cell 2014; 26: 565–576. 25220446 10.1016/j.ccr.2014.07.026

[15] Urban EM , Chapoval AI , Pauza CD . Repertoire development and the control of cytotoxic/effector function in human γδ T cells. Clin Dev Immunol 2010; 2010: 732893. 20396597 10.1155/2010/732893 PMC2854522

[16] Ravens S , Schultze‐Florey C , Raha S *et al* Human γδ T cells are quickly reconstituted after stem‐cell transplantation and show adaptive clonal expansion in response to viral infection. Nat Immunol 2017; 18: 393–401. 28218745 10.1038/ni.3686

[17] Bosch M , Khan FM , Storek J . Immune reconstitution after hematopoietic cell transplantation. Curr Opin Hematol 2012; 19: 324–335. 22517587 10.1097/MOH.0b013e328353bc7d

[18] Silva‐Santos B , Mensurado S , Coffelt SB . γδ T cells: pleiotropic immune effectors with therapeutic potential in cancer. Nat Rev Cancer 2019; 19: 392–404. 31209264 10.1038/s41568-019-0153-5 PMC7614706

[19] Willcox CR , Davey MS , Willcox BE . Development and selection of the human Vγ9Vδ2^+^ T‐cell repertoire. Front Immunol 2018; 9: 1501. 30013562 10.3389/fimmu.2018.01501 PMC6036166

[20] Farnault L , Gertner‐Dardenne J , Gondois‐Rey F *et al* Clinical evidence implicating γδ T cells in EBV control following cord blood transplantation. Bone Marrow Transplant 2013; 48: 1478–1479. 23832093 10.1038/bmt.2013.75

[21] Bian Z , Liu J , Xu LP *et al* Association of Epstein‐Barr virus reactivation with the recovery of CD4/CD8 double‐negative T lymphocytes after haploidentical hematopoietic stem cell transplantation. Bone Marrow Transplant 2017; 52: 264–269. 27797369 10.1038/bmt.2016.238

[22] Liu J , Bian Z , Wang X *et al* Inverse correlation of Vδ2^+^ T‐cell recovery with EBV reactivation after haematopoietic stem cell transplantation. Br J Haematol 2018; 180: 276–285. 29270985 10.1111/bjh.15037

[23] Armand P , Kim HT , Logan BR *et al* Validation and refinement of the Disease Risk Index for allogeneic stem cell transplantation. Blood 2014; 123: 3664–3671. 24744269 10.1182/blood-2014-01-552984 PMC4047501

[24] Ando T , Tachibana T , Tanaka M *et al* Impact of graft sources on immune reconstitution and survival outcomes following allogeneic stem cell transplantation. Blood Adv 2020; 4: 408–419. 31990335 10.1182/bloodadvances.2019001021 PMC6988395

[25] Bejanyan N , Brunstein CG , Cao Q *et al* Delayed immune reconstitution after allogeneic transplantation increases the risks of mortality and chronic GVHD. Blood Adv 2018; 2: 909–922. 29678809 10.1182/bloodadvances.2017014464 PMC5916001

[26] Mehta RS , Rezvani K . Immune reconstitution post allogeneic transplant and the impact of immune recovery on the risk of infection. Virulence 2016; 7: 901–916. 27385018 10.1080/21505594.2016.1208866 PMC5160395

[27] Kanakry CG , Ganguly S , Zahurak M *et al* Aldehyde dehydrogenase expression drives human regulatory T cell resistance to posttransplantation cyclophosphamide. Sci Transl Med 2013; 5: 211ra157. 10.1126/scitranslmed.3006960 PMC4155575 24225944

[28] Roberto A , Castagna L , Zanon V *et al* Role of naive‐derived T memory stem cells in T‐cell reconstitution following allogeneic transplantation. Blood 2015; 125: 2855–2864. 25742699 10.1182/blood-2014-11-608406 PMC4424633

[29] Kanakry CG , Coffey DG , Towlerton AM *et al* Origin and evolution of the T cell repertoire after posttransplantation cyclophosphamide. JCI Insight 2016; 1: e86252. 27213183 10.1172/jci.insight.86252 PMC4874509

[30] Retiere C , Willem C , Guillaume T *et al* Impact on early outcomes and immune reconstitution of high‐dose post‐transplant cyclophosphamide vs anti‐thymocyte globulin after reduced intensity conditioning peripheral blood stem cell allogeneic transplantation. Oncotarget 2018; 9: 11451–11464. 29545911 10.18632/oncotarget.24328 PMC5837739

[31] Malard F , Labopin M , Chevallier P *et al* Larger number of invariant natural killer T cells in PBSC allografts correlates with improved GVHD‐free and progression‐free survival. Blood 2016; 127: 1828–1835. 26903546 10.1182/blood-2015-12-688739

[32] Arruda LCM , Gaballa A , Uhlin M . Impact of γδ T cells on clinical outcome of hematopoietic stem cell transplantation: systematic review and meta‐analysis. Blood Adv 2019; 3: 3436–3448. 31714966 10.1182/bloodadvances.2019000682 PMC6855117

[33] Nielsen MM , Witherden DA , Havran WL . γδ T cells in homeostasis and host defence of epithelial barrier tissues. Nat Rev Immunol 2017; 17: 733–745. 28920588 10.1038/nri.2017.101 PMC5771804

[34] Dulery R , Menard AL , Chantepie S *et al* Sequential conditioning with thiotepa in T cell‐ replete hematopoietic stem cell transplantation for the treatment of refractory hematologic malignancies: comparison with matched related, haplo‐mismatched, and unrelated donors. Biol Blood Marrow Transplant 2018; 24: 1013–1021. 29337223 10.1016/j.bbmt.2018.01.005

[35] Malard F , Szydlo RM , Brissot E *et al* Impact of cyclosporine‐A concentration on the incidence of severe acute graft‐versus‐host disease after allogeneic stem cell transplantation. Biol Blood Marrow Transplant 2010; 16: 28–34. 20053329 10.1016/j.bbmt.2009.08.010

[36] Stocker N , Dulery R , Battipaglia G *et al* Impact of cyclosporine A concentration on acute graft‐vs‐host disease incidence after haploidentical hematopoietic cell transplantation. Eur J Haematol 2019; 103: 10–17. 30958904 10.1111/ejh.13233

[37] Przepiorka D , Weisdorf D , Martin P *et al* 1994 consensus conference on acute GVHD grading. Bone Marrow Transplant 1995; 15: 825–828. 7581076

[38] Shulman HM , Sullivan KM , Weiden PL *et al* Chronic graft‐versus‐host syndrome in man. A long‐term clinicopathologic study of 20 Seattle patients. Am J Med 1980; 69: 204–217. 6996481 10.1016/0002-9343(80)90380-0

[39] Ruggeri A , Labopin M , Ciceri F , Mohty M , Nagler A . Definition of GvHD‐free, relapse‐free survival for registry‐based studies: an ALWP‐EBMT analysis on patients with AML in remission. Bone Marrow Transplant 2016; 51: 610–611. 26657834 10.1038/bmt.2015.305

