# Supplementary material for: High‐dose post‐transplant cyclophosphamide impairs γδ T‐cell reconstitution after haploidentical haematopoietic stem cell transplantation using low‐dose antithymocyte globulin and peripheral blood stem cell graft
Source: Clin Transl Immunology. 2020 Sep 23;9(9):e1171. doi: 10.1002/cti2.1171 (PMC7511259; doi:10.1002/cti2.1171)
Supplement: Supplementary file 1 [file CTI2-9-e1171-s001.docx]

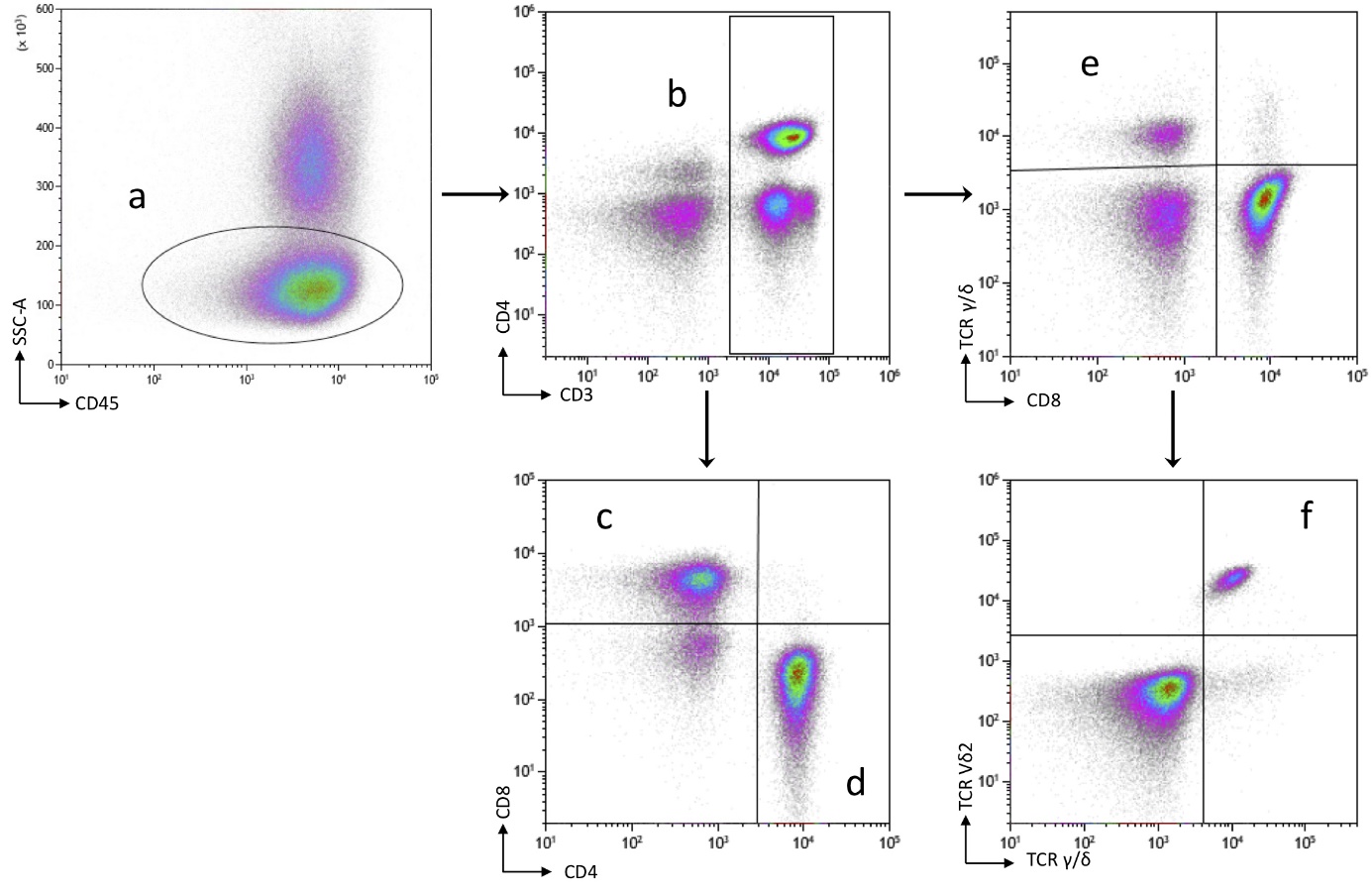


**Supplementary figure 1. Gating strategy for analysis of T cell subpopulations.**

Gating of lymphocytes was sequentially done on CD45^+^ and low SSC-A (**1a**). CD3^+^ were selected (**1b**) and CD8^+^ (**1c**) and CD4^+^ (**1d**) cells were discriminated according to the expression of CD8 and CD4 in CD3^+^ cells. Finally, γ/δ T cells were selected on CD3^+^ cells for their positivity for TCR γ/δ (**1e**) and Vδ2^+^ T cells were selected on γ/δ T cells for their positivity for TCR Vδ2 (**1f**), respectively.
