# Supplementary material for: High‐dose post‐transplant cyclophosphamide impairs γδ T‐cell reconstitution after haploidentical haematopoietic stem cell transplantation using low‐dose antithymocyte globulin and peripheral blood stem cell graft
Source: Clin Transl Immunology. 2020 Sep 23;9(9):e1171. doi: 10.1002/cti2.1171 (PMC7511259; doi:10.1002/cti2.1171)
Supplement: Supplementary file 2 [file CTI2-9-e1171-s002.docx]

**Supplementary figure 2. Immune recovery of T cells after Allo-HCT in patients receiving cyclosporine A and mycophenolate mofetil**

##### Kinetics of absolute lymphocyte (1a), T cell (1b), CD4^+^ T cell (1c), CD8^+^ T cell (1d), γ/δ T cell (1e) and Vδ2^+^ T cell (1f) counts in blood from control (n= 64) and Haplo-HCT with PTCy (n= 19) recipients following Allo-HCT. Samples from healthy donors serve as baseline reference. Box and whisker plots displaying the median, the 25th percentile, the 75th percentile of the distribution (box), and the most extreme data point (whiskers), which is no more than 1.5 times the interquartile range from the box. CsA, cyclosporine A; MMF, mycophenolate mofetil; PTCy, post-transplant cyclophosphamide; Allo-HCT, allogeneic hematopoietic stem cell transplantation; HD, healthy donors.
