# Supplementary material for: High‐dose post‐transplant cyclophosphamide impairs γδ T‐cell reconstitution after haploidentical haematopoietic stem cell transplantation using low‐dose antithymocyte globulin and peripheral blood stem cell graft
Source: Clin Transl Immunology. 2020 Sep 23;9(9):e1171. doi: 10.1002/cti2.1171 (PMC7511259; doi:10.1002/cti2.1171)
Supplement: Supplementary file 4 [file CTI2-9-e1171-s004.docx]

**Supplementary table 1. Immune reconstitution after allogeneic hematopoietic stem cell transplantation**

| Immune subset (number μl^-1^) | Control group (n=87) | PTCy group (n=19) | *P*-value |
| --- | --- | --- | --- |
| Total lymphocytes – median (range) |  |  |  |
| D20 | 360 (30-1380) | 100 (10-420) | **< .0001** |
| D30 | 525 (30-2260) | 380 (0-1250) | 0.13 |
| D90 | 560 (40-1890) | 590 (50-1140) | 0.96 |
| D180 | 865 (160-3640) | 810 (60-2480) | 0.75 |
| D360 | 1120 (60-3790) | 1375 (220-3860) | 0.51 |
| CD3^+^ T cells – median (range) |  |  |  |
| D20 | 88 (0-1090) | 8 (0-190) | **< .0001** |
| D30 | 156 (1-1556) | 148 (0-564) | 0.20 |
| D90 | 234 (9-1337) | 235 (0-804) | 0.72 |
| D180 | 466 (45-1719) | 318 (22-2178) | 0.30 |
| D360 | 492 (5-2732) | 875 (62-3541) | 0.15 |
| CD3^+^CD4^+^ T cells – median (range) |  |  |  |
| D20 | 11 (0-264) | 1 (0-24) | **< .0001** |
| D30 | 27 (0-792) | 19 (0-113) | 0.06 |
| D90 | 63 (4-409) | 47 (0-194) | 0.34 |
| D180 | 105 (5-608) | 112 (13-203) | 0.87 |
| D360 | 123 (1-603) | 182 (30-705) | 0.57 |
| CD3^+^CD8^+^ T cells – median (range) |  |  |  |
| D20 | 40 (0-880) | 4 (0-163) | **< .0001** |
| D30 | 68 (0-1105) | 57 (0-286) | 0.30 |
| D90 | 122 (3-850) | 127 (0-536) | 0.90 |
| D180 | 212 (29-1182) | 132 (4-1932) | 0.37 |
| D360 | 214 (1-1785) | 484 (8-2784) | 0.29 |
| γ/δ T cells – median (range) |  |  |  |
| D20 | 6 (0-127) | 0 (0-5) | **< .0001** |
| D30 | 10 (0-406) | 2 (0-156) | **0.002** |
| D90 | 15 (0-239) | 5 (0-217) | 0.29 |
| D180 | 19 (1-328) | 8 (1-156) | 0.06 |
| D360 | 24 (0-352) | 25 (2-136) | 0.48 |
| Vδ2^+^ T cells – median (range) |  |  |  |
| D20 | 1 (0-116) | 0 (0-3) | **< .0001** |
| D30 | 5 (0-125) | 0 (0-150) | **0.0005** |
| D90 | 5 (0-87) | 0 (0-211) | **0.0005** |
| D180 | 10 (0-116) | 1 (0-155) | **0.009** |
| D360 | 10 (0-293) | 3 (0-112) | **0.02** |

D, day; PTCy, post-transplant cyclophosphamide.

Bold denotes statistically significant.
