# Supplementary material for: High‐dose post‐transplant cyclophosphamide impairs γδ T‐cell reconstitution after haploidentical haematopoietic stem cell transplantation using low‐dose antithymocyte globulin and peripheral blood stem cell graft
Source: Clin Transl Immunology. 2020 Sep 23;9(9):e1171. doi: 10.1002/cti2.1171 (PMC7511259; doi:10.1002/cti2.1171)
Supplement: Supplementary file 5 [file CTI2-9-e1171-s005.docx]

**Supplementary table 2. Cohort 2 population and transplant characteristics**

| Characteristics | Control group  (n=16) | PTCy group  (n=12) | *P*-value |
| --- | --- | --- | --- |
| Patient age, (yrs) median (range) | 63 (19-74) | 64 (29-74) | 0.40 |
| Patient gender |  |  |  |
| Male | 7 (44) | 6 (50) | 1 |
| Female | 9 (56) | 6 (50) |  |
| Donor gender |  |  |  |
| Female to male | 3 (19) | 1 (8) | 0.61 |
| CMV seronegative donor-recipient pair | 0 | 3 (25) | 0.07 |
| EBV seronegative donor-recipient pair | 0 | 0 | - |
| Diagnosis |  |  |  |
| Myeloid malignancies | 13 (82) | 11 (92) | 0.61 |
| Acute myeloid leukemia | 10 (63) | 8 (67) |  |
| Myeloproliferative neoplasms | 1 (6) | 2 (17) |  |
| Myelodysplastic syndrome | 2 (13) | 1 (8) |  |
| Lymphoid malignancies | 3 (18) | 1 (8) |  |
| Acute lymphoblastic leukemia | 1 (6) | 1 (8) |  |
| Non-Hodgkin lymphoma | 1 (6) | 0 |  |
| Chronic lymphocytic leukemia | 1 (6) | 0 |  |
| Disease Risk Index |  |  |  |
| Low | 1 (6) | 0 | 0.90 |
| Intermediate | 8 (50) | 5 (42) |  |
| High | 7 (44) | 6 (50) |  |
| Very High | 0 | 1 (8) |  |
| Conditioning regimen category |  |  |  |
| RIC | 0 | 1 (8) | 0.60 |
| RTC | 14 (88) | 9 (75) |  |
| Sequential | 2 (12) | 2 (17) |  |
| Post-transplant immunosuppression |  |  |  |
| CsA alone | 9 (56) | 0 | **< .0001** |
| CsA and MMF | 7 (44) | 0 |  |
| CsA and MMF and PTCy | 0 | 12* (100) |  |

CMV, cytomegalovirus; EBV, Epstein-Barr virus, RIC, reduced-intensity conditioning; RTC; reduced-toxicity conditioning CsA, cyclosporine A; MMF, mycophenolate mofetil; PTCy, post-transplant cyclophosphamide.

* PTCy 50 mg/kg/day at D3 and D5.

Bold denotes statistically significant.
