# Supplementary material for: High‐dose post‐transplant cyclophosphamide impairs γδ T‐cell reconstitution after haploidentical haematopoietic stem cell transplantation using low‐dose antithymocyte globulin and peripheral blood stem cell graft
Source: Clin Transl Immunology. 2020 Sep 23;9(9):e1171. doi: 10.1002/cti2.1171 (PMC7511259; doi:10.1002/cti2.1171)
Supplement: Supplementary file 6 [file CTI2-9-e1171-s006.docx]

**Supplementary table 3. Univariate analysis of EBV risk factors**

| Variates | EBV incidence at month 12, % (95%CI) | *P*-value |
| --- | --- | --- |
| CD3^+^ T cells at day 30  ≤ 154.1 μL^-1^  > 154.1 μL^-1^ | 40 (24-55)  34 (19-49) | 0.44 |
| CD4^+^ T cells at day 30  ≤ 11.62 μL^-1^  > 11.62 μL^-1^ | 25 (10-44)  42 (28-55) | 0.13 |
| CD8^+^ T cells at day 30  ≤ 6.81 μL^-1^  > 6.81 μL^-1^ | 55 (20-79)  34 (22-45) | 0.27 |
| γ/δ T cells at day 30  ≤ 4.63 μL^-1^  > 4.63 μL^-1^ | 53 (34-70)  26 (14-39) | **0.006** |
| Vδ2^+^ T cells at day 30  ≤ 0.49 μL^-1^  > 0.49 μL^-1^ | 67 (35-86)  29 (18-41) | **0.003** |
| Conditioning regimen  RTC/sequential  RIC | 42 (31-52)  21 (5-46) | 0.10 |
| Patient age  ≤ median  > median | 32 (20-45)  46 (32-60) | 0.25 |
| Disease Risk Index  Low/Intermediate  High/Very high | 36 (23-50)  42 (28-55) | 0.75 |
| PTCy  Yes  No | 61 (34-80)  34 (24-45) | **0.02** |

CI, confidence interval; EBV, Epstein-Barr virus; RIC, reduced-intensity conditioning; RTC, reduced-toxicity conditioning PTCy, post-transplant cyclophosphamide.

Bold denotes statistical significance.
